# From robot to molecule, the behavior

**DOI:** 10.1590/S1677-5538.IBJU.2016.03.02

**Published:** 2016

**Authors:** Julio Bernardes

**Affiliations:** Prof. Adjunto de Urologia - UFPA (Universidade Federal do Pará). Discente de doutorado do PPGOCM - Núcleo de Pesquisas em Oncologia / UFPA. Hospital Universitário João de Barros Barreto, 2º Piso da UNACON. Rua dos Mundurucus, 4487, Guamá. Belém, PA, 66073-005, Brasil. E-mail: juliobernardes@oi.com.br

In general Urology, forty per cent of ambulatory consultations are intended to prostate care, as well as consultation in geriatrics and clinical medicine. It is observed statistically that one in every six men over 50 years will present prostate cancer (PCa) throughout live (1). It is the second most prevalent cancer in men, following skin cancer, with an estimate of 61,200 new cases in 2016, according to INCA (National Cancer Institute of Brazil).

Recent scientific knowledge and incorporation of new technologies lead to higher interaction with molecular epidemiology and cancer genetics. They explain why some patients will present slower progression of the disease, allowing for active surveillance, and also the use of newer and lesser aggressive treatments with higher survival with good quality of life. Epigenetic and genetic alterations provide a mosaic of tumor clones that determine respectively heterogeneous histologically phenotypic tumors, with corresponding indolent clinical symptoms or a more aggressive progression (2).

Currently, renal tumors are efficiently treated due to precision and richer details provided by modern image technologies. We are able to detect in daily practice the aggressiveness of the lesion according to dimension, morphology, tissue density, perfusion and anatomic relations, allowing the choice of the most adequate treatment. Actually, current image exams reflect more accurately the tumor microenvironment. In the same way, evaluation of prostate gland by multiparametric magnetic resonance provides data related to morphology, perfusion, diffusion and spectroscopy, that matches more adequately tumor histology and neoplastic alterations of PCa. After 2010, based on the BIS-RADS model system, Breast Imaging and Reporting Archiving Data System, many studies have been proposed to study the prostate gland. PI-RADS, Prostate Imaging and Reporting Archiving Data System, was proposed to determine image patterns obtained by MRI of PCa, in order to distinguish between “insignificant lesions” and clinical significant lesions, and to determine where to perform biopsy (prostate targets). In 2012 European Urology magazine proposed a guideline using PI-RADS system with five grades of suspicion of prostate cancer. In the first two grades, it is unlikely the presence of clinical significant disease and biopsy is not recommended; grade three is undetermined and the last two grades present respectively increased rate of predictive value/positivity of prostate cancer, determining the need of prostate biopsy (3). This method of prostate evaluation presents an intrinsic correlation of histopathological findings according to Gleason system and morphological and functional images classified according to PI-RADS system, related to the molecular content of tumor cells. This classification allows for therapeutic variations, from active surveillance to minimally invasive focal ablations or radical surgeries and expanded lymphadenectomy. The better understanding of cellular signal alterations of prostate cancer resulted in the development of new treatments, such as the new generation of anti-androgens.

In a similar way, Gleason system has been modified over the years since its first publication. It is a morphological and analogical system fundamental to diagnostic, prognostic and treatment of prostate cancer. In November 2014 a new recommendation of International Society of Urological Pathology (ISUP), proposed the grouping of scores in five categories (4), based on the recognition that previous score valued some benign lesions (Table-1).

**Table 1:** New recommendation of International Society of Urological Pathology (ISUP) (4).

Grade I	Score 3 + 3
Grade II	Score 3 + 4
Grade III	Score 4 + 3
Grade IV	Score 8 (3 + 5, 5 + 3 and 4 + 4)
Grade V	Score 9 and 10 (4 + 5, 5 + 4 and 5 + 5)

This valuable system of morphological classification of prostate tissue since the beginning showed the heterogeneity of tumor histological findings present in the same gland with obvious different biological behavior and distinct evolution according to focus, making treatment approach complex. These variations of PCa histology are being scientifically endorsed, correlating each grade of Gleason scale with a respective profile of genic expressions, related to a specific assortment of carcinogenic cell signals, that will act as tumor progression markers. Welsh et al work described 20 genes with different expressions correlated to three grades of Gleason score. Insulin-binding proteins (IGFRP 2 and 5) were expressed in higher grade tumors (5).

Current urological practice is guided by a clinical rationale based on molecular biology of PCa, and urologists, pathologists and oncologists apply laboratory research data and clinical daily practice evidences in clinical treatments.

In the current treatment of our patients, it is mandatory to understand proliferation and cellular differentiation according to epigenetics, cellular cycle regulations and possible alterations of signalization among androgens, co-activators and androgen receptors.

Many years have passed in order to aware global male population about the importance of early diagnosis of prostate cancer, with unquestionable positive results. But current prostate cancer screening methods are controversial and maybe the explanation of these troublesome epidemiological polemics is based on the PCa heterogeneity including molecular aspects and familiar history; the understanding of those aspects may help us redirect PCa screening.

For many years, it has been shown that first degree relatives with PCa and relatives with breast cancer with less than 36 years old increase four-fold the chance of PCa. Five to 10% of all cancer are hereditary transmitted by mutations that occurred in germ cells, being defined as constitutional tumors. Hereditary cancer usually presents more clinical and aggressive evolution. It is mandatory to have in mind that individuals are born frequently with loss of one of two tumor genic suppressor activity alleles. Consequently, timing of phenotypically expression of tumor is shortened when the remaining “health’ allele loses its function. This is the concept that explain predisposing syndromes of cancer, and typical examples of hereditary cancer are breast and colon tumors. Although it is not described a specific characterization of hereditary prostate cancer, others syndromes of hereditary tumors that include PCa are known (6). Also, PCa presents a great number of studied polymorphisms that explains the genesis of PCa (common genetic alteration of general population that may predispose to tumor (6, 7)) (Table-2).

**Table 2:** Genes more involved in PCa (6, 7).

Gene	Gene	Gene
DAB2IP	HERC2	LEPR
IL4	RNASEL	CRY
ARCF	HOXB13	OGGI
HPC1	HPC2	MSR1
PON1	MIC1	BRCA1 / BRCA2

It is recommended during anamnesis to construct a heredogram in men over 40 years old with at least three generations by which it is possible to choose individual preventive measures mainly for hereditary syndrome of breast and ovary cancer (HBOC) whose sites are closely related to PCa. Hereditary tumors usually present some of the following characteristics (8):

1. Increase number of cases in a particular population
2. Multiple cases in the same family, involving many generations
3. Bilateral tumors, or more than one primary tumor in the same individual, synchronous or metachronous
4. Rare histological type of tumors
5. Cases in younger age than in general population

Currently, we observe the duel between professional performance and surgical technique of robotic laparoscopic radical prostatectomy compiling results and complications, and each urologist is invited to analyze his personal limits and personal skills. Also, the urologists are presented with several forms of treatment, from resection of advanced tumors to simple clinical observation and the use of new drugs that interact with molecular signs, such as abiraterone and enzalutamide and he must adequately understand molecularly the phenomena and their clinical consequences.

In conclusion, it is important to recognize and understand that molecules determine distinct biological behaviors and when we are able to identify and assimilate different molecular profiles we will be capable to practice a precision medicine, with adequate treatment of our patients, from simple surveillance to robotic surgery.

Julio Bernardes, MD, PhD Prof. Adjunto de Urologia - UFPA (Universidade Federal do Pará) Discente de doutorado do PPGOCM - Núcleo de Pesquisas em Oncologia / UFPA Hospital Universitário João de Barros Barreto, 2º Piso da UNACON Rua dos Mundurucus, 4487, Guamá Belém, PA, 66073-005, Brasil E-mail: juliobernardes@oi.com.br
